# Therapeutic Effects of Fucoidan: A Review on Recent Studies

**DOI:** 10.3390/md17090487

**Published:** 2019-08-21

**Authors:** Sibusiso Luthuli, Siya Wu, Yang Cheng, Xiaoli Zheng, Mingjiang Wu, Haibin Tong

**Affiliations:** College of Life and Environmental Science, Wenzhou University, Wenzhou 325035, China

**Keywords:** fucoidan, therapeutic effects, bioactivity, anti-viral

## Abstract

Fucoidan is a polysaccharide largely made up of l-fucose and sulfate groups. Fucoidan is favorable worldwide, especially amongst the food and pharmaceutical industry as a consequence of its promising therapeutic effects. Its applaudable biological functions are ascribed to its unique biological structure. Classical bioactivities associated with fucoidan include anti-oxidant, anti-tumor, anti-coagulant, anti-thrombotic, immunoregulatory, anti-viral and anti-inflammatory effects. More recently, a variety of in vitro and in vivo studies have been carried out to further highlight its therapeutic potentials. This review focuses on the progress towards understanding fucoidan and its biological activities, which may be beneficial as a future therapy. Hence, we have summarized in vitro and in vivo studies that were done within the current decade. We expect this review and a variety of others can contribute as a theoretical basis for understanding and inspire further product development of fucoidan.

## 1. Introduction

The marine environment is renowned as a rich source of chemical and biological diversity. This type of diversity has been regarded as a unique source of chemical compounds for cosmetics, dietary supplementation, agrochemicals and pharmaceuticals [1]. Seaweeds, such as green algae, red algae and brown algae, are able to produce various metabolites characterized by a broad spectrum of biological activities [2]. A number of studies have been conducted towards their nutraceutical and pharmaceutical properties [3,4,5].

For about a period of 2000 years, brown algae, such as *Sargassum* spp., has been put to use as traditional Chinese medicine (TCM) towards treating various diseases, including thyroid diseases such as goiters [6]. In addition, it has also been used traditionally to treat scrofula, tumors, edema, testicular pain, swelling, cardiovascular diseases, arteriosclerosis, ulcer, renal issues, eczema, scabies, psoriasis and asthma [6]. Their therapeutic effects have been scientifically approved and may, therefore, be explained by means of in vivo and in vitro pharmacological activities, such as producing anti-cancer, anti-inflammatory, anti-bacterial, anti-viral, neuroprotective and anti-HIV activities.

Several studies and reviews have been done in the past on the bioactivity of fucoidan e.g., by producing anti-oxidant, anti-tumor, immunoregulation, anti-viral and anti-coagulant activities [7]. Our aim is to cover a variety of angles on the contributing factors behind the bioactivities of fucoidan, such as their source, molecular weight (Mw), sulfate group and extraction methods. In addition, we did a follow up on some studies done by certain groups with the aim to observe and compare progress based on their previous studies.

## 2. Summary of The Literature

It seems evident that fucoidan is gaining interest as a potential therapy, which is part of the highlight of this literature. Though this may be the case, a lot of ground still needs to be covered, first by understanding the concept, origin and source of fucoidan (Section 3 and Section 4). One of the most important points in this review is the structure and pharmacokinetics of fucoidan, which sort of gives us a general idea on how the mechanism of bioactivity gets executed by fucoidan, especially when it comes to its structure and pharmacokinetics (Section 5 and Section 6). Since fucoidan is identified as possessing pro-apoptotic abilities, we summarized studies that were stimulated by fucoidan—for instance in cancers. We also reflected upon different mechanisms within the process of apoptosis e.g., cell-cycle arrest, intrinsic and extrinsic pathways (Section 7). Section 8, Section 9, Section 10 and Section 11 provides a summary of the anti-viral activities of fucoidan, including anti-influenza virus, anti-hepatitis B, anti-HIV and anti-canine distemper virus (CDV). In the past, a variety of studies have shown fucoidan’s potential as possessing anti-diabetic capabilities. Some in vitro studies have characterized fucoidan’s ability to reverse the classical symptoms of diabetes and related metabolic syndromes, and we have provided a summary of these characterizations in Section 12. Plants of marine origin, such as fucoidan, based on previous literature are also regarded as an anti-coagulant, hence it was suitable to include such a summary in Section 13. Therefore, the aim was to use an all-round approach into the health benefits (e.g., anti-cancer, anti-viral, anti-coagulant, anti-diabetic) that are generally mentioned by authors who focus their studies of fucoidan, while looking into the structure and characteristics of fucoidan.

## 3. Fucoidan

The first extraction of fucoidan was in 1913 from a species of brown algae [8], such as *Laminaria digitata*, *Ascophyllum nodosum* and *Fucus vesiculosus*. Fucoidan is a negatively charged and highly hygroscopic polysaccharide [9]. A high content of fucoidan is mainly found in the leaves of *L. digitata*, *A. nodosum*, *Macrocystis pyrifera* and *F. vesiculosus*. Fucoidan is soluble in both water and acid solutions. After the first publication took place in 1913, the number of published articles (studies) on fucoidan has increased significantly, especially in the modern era. The reason behind the increase in studies is that fucoidan has anti-tumor, anti-coagulant and anti-oxidant activities, as well as the importance in terms of regulating the metabolism of glucose and cholesterol [10]. Also, there has been an interest in fucoidan because of its potential to provide protection against liver damage and urinary system failures. It is evident that research on fucoidan is gradually flourishing, as these activities are carried out, and more of its bio-activities and health-related benefits are being discovered as studies continue to accumulate.

## 4. Sources of Fucoidan

**Figure 1:**
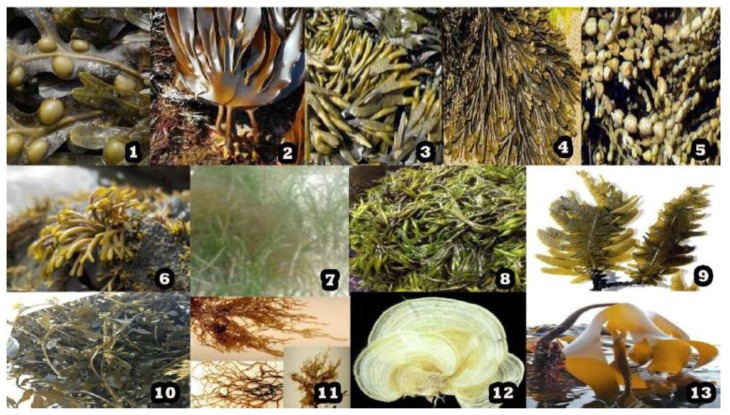
Sources of fucoidan. 1. *Fucus vesiculosus*, 2. *Laminaria digitata*, 3. *Fucus evanescens*, 4. *Fucus serratus*, 5. *Ascophyllum nodosum*, 6. *Pelvetia canaliculata*, 7. *Cladosiphon okamuranus*, 8. *Sargassum fusiforme*, 9. *Laminaria japonica*, 10. *Sargassum horneri*, 11. *Nemacystus decipiens*, 12. *Padina gymnospora*, 13. *Laminaria hyperborea*.

Fucoidan is a sulfated polysaccharide which can be found amongst a number of marine sources, including sea cucumbers [11] or brown algae [12]. A great number of algae and invertebrates have been ascertained for their fucoidan contents inclusive of *Fucus vesiculosus*, *Sargassum stenophyllum*, *Chorda filum*, *Ascophyllum nodosum*, *Dictyota menstrualis*, *Fucus evanescens*, *Fucus serratus*, *Fucus distichus*, *Caulerpa racemosa*, *Hizikia fusiforme*, *Padina gymnospora*, *Kjellmaniella crassifolia*, *Analipus japonicus* and *Laminaria hyperborea* exhibited in Figure 1. In these sources, different types of fucoidan can be obtained and the methods of extraction employed are different, especially when they are reported in different studies.

## 5. Structure of Fucoidan

**Figure 2:**
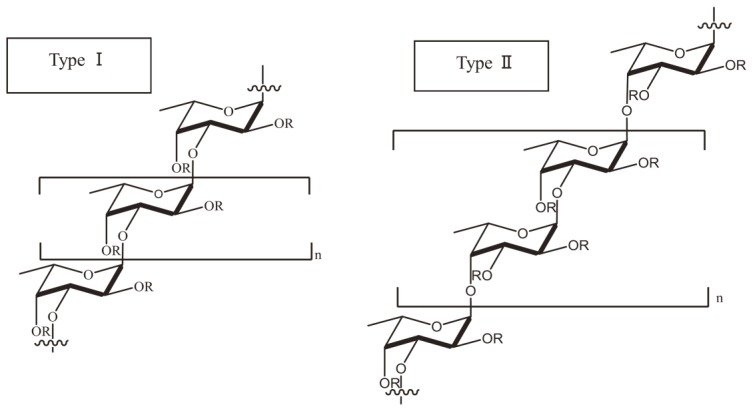
Type I and type II of common backbone chains in brown seaweed fucoidan. R can be fucopyranose, glucuronic acid and sulfate groups, while the location of galactose, mannose, xylose, rhamnose, arabinose and glucose in several kinds of seaweed species remains unknown.

Fucoidan is known as a fucose-enriched and sulfated polysaccharide that is mainly sourced from the extracellular matrix of brown algae. Fucoidan is made up of l-fucose, sulfate groups and one or more small proportions of xylose, mannose, galactose, rhamnose, arabinose, glucose, glucuronic acid and acetyl groups in a variety of brown algae [13,14,15]. In a number of studies, researchers have also used galactofucan to represent a kind of fucoidan. Galactofucan is known as a monosaccharide, and the composition of the monosaccharide is galactose accompanied by fucose, similar to rhamnofucan (rhamnose and fucose) and rhamnogalactofucan (rhamnose, galactose and fucose). In addition to the structure of fucoidan, there is also a variation amongst different seaweed types. Nevertheless, fucoidan normally has two types of homofucose (Figure 2). One type (I) encompasses repeated (1→3)-l-fucopyranose, and the other type (II) encompasses alternating and repeated (1→3)- and (1→4)-l-fucopyranose [16].

Reports based on structures of fucoidan, sourced from different species of brown algae, brought about an improved categorization in terms of structures. By a way of illustration, most of the fucoidans sourced from species belonging to the *Fucales* have an alternating linkage of (1→3)-α-l-fucose and (1→4)-α-l-fucose [17,18,19,20,21]. Structures of *Ascophyllum nodosum* fucoidan [22] and *F. vesiculosus* fucoidan show a resemblance of one another, the difference is only significant based on sulfate patterns and the presence of glucuronic acid. A number of *Fucales* species, such as *Fucus serratus*, *Fucus distichus* and *Pelvetia canaliculate*, present similar fucoidan backbone, but show more diversity in the branching and the presence of different monosaccharides [20,21,23]. However, exceptions do exist, for instance, fucoidans from the *Bifurcaria bifucardia* and *Himanthalia elongate* do not follow or ascribe to such a structural feature [24]. Hence, identifying the structure of fucoidan based on the species they belong to presents a challenge.

Another important fact that deserves mentioning is that the structure of fucoidan is also highly dependent on the harvest season. This is based on the *Undaria pinnafida* fucoidan, which exhibited distinct characteristics and bioactivity, especially when harvested during different seasons [25,26]. In addition, it should be indicated that the purification method also plays a critical role in the structure of fucoidan. To such an extent that new purification methods have led to the revelation that the fucoidan structure is comprised of multiple fractions [27]. An investigation reported that the structure of crude fucoidan sourced from *A. nodosum* showed a predominant repetition of [→(3)-α-l-Fuc(2SO_3_^−^) − (1→4)-α-Fuc(2,3diSO_3_^−^)-(1)]n [28]. However, from the same species, a purified fraction comprised of primarily α-(1→3)-fucosyl residues with a sparse linkage of α-(1→4) and was found to be highly branched [29]. Therefore, the employment of different extraction methods results in distinct structures. For example, a report states that one species produced two distinct fucoidan structures, particularly galactofuctans and uronofucoidans [30]. Hence, it should be emphasized that purification techniques are one of the determining factors towards the structure and the associated bioactivities.

## 6. Pharmacokinetics Research of Fucoidan

It can be mentioned that quite a few experimental activities have been undertaken to address the so-called ADME i.e., absorption, distribution, metabolism and excretion of fucoidan. The confirmation of fucoidan absorption was determined using ELISA with fucoidan-specific antibody [31,32,33]. An absorption study was performed on rats using *F. vesiculosus* fucoidan (737 kDa), while 4 h after administration, a maximum concentration of fucoidan in serum was reached, which then resulted in the accumulation of the absorbed fucoidan in the kidneys. The fucoidan accumulation in organs has also been confirmed by the absorption of *C. okamuranus* fucoidan in rats [34]. In addition, authors’ observation from healthy volunteers who either orally ingested or were administered with fucoidan showed that some portion of fucoidan was absorbed by means of endocytosis, and was detected both in their serum and urine [35]. It could be mentioned that LMWF (low molecular weight fucoidan) may be further developed to be used for clinical purposes. This is in relation to a comparative investigation (i.e., LMWF and MMWF (middle molecular weight fucoidan) from *S. japonica*), the outcome was that LMWF presented with a better absorption rate and bioavailability, hence supporting its biological potential [36]. However, fucoidan may still present with favorable pharmacokinetics in relation to toxicity; the information on its biodistribution in human is still insufficient. Animal models indicate its bioavailability, sparking interest toward the LWMF as a potential solution. The latest study by Kadena et al. [37] pursued a slightly different approach when investigating the absorption of fucoidan based on oral administration. They concluded that “the habit of eating mozuku was speculated to be a factor in the absorption of fucoidan”. A total of 396 volunteers were investigated after they ingested 3 g of mozuku fucoidan, fucoidan was detected in the urine specimens of the 385 participants out of the 396. Hence, in addition to the conclusion, participants residing in the location of Okinawa presented with increased urinary excretion of fucoidan, because the locals of the Okinawa region generally consumed greater amounts of mozuku than those outside the region. Although further studies on absorption across the intestinal tract should be performed, it is rather interesting to have scientists pursuing different angles towards fucoidan absorption. Hence, future developments of fucoidan as a drug will be based on well-informed choices, due to a wide range of information that is gradually accumulating.

To date, two clinical trials are underway, such trials are focused on the biodistribution and tolerance of fucoidan. Healthy individuals or volunteers are engaged in tests that involve the biodistribution, safety and dosimetry of a labeled fucoidan (ClinicalTrials.gov, Identifier: NCT03422055). In another trial, patients with stage III-IV non-small cell lung cancer (NSCLC) are being studied (in a placebo-controlled trial), whereby fucoidan is added to their chemotherapy treatment to determine the impact it would present on their quality of life (ClinicalTrials.gov, Identifier: NCT03130829). The results of these studies (clinical trials) will play an important role in gaining insight in ADME and toxicity of fucoidan in human beings.

## 7. Anti-Cancer Capacity

Apoptosis is a physiological process that is known as programmed cell death and is essential for embryonic development and homeostasis in organisms, but it can also participate in pathological processes, e.g., cancer [38]. Therefore, this section follows up on how malignant or cancer cells undergo apoptosis after the administration or stimulation by fucoidan, in different manners, i.e., caspases, cell cycle arrest, intrinsic and extrinsic pathways. *C. okamuranus* fucoidan (average Mw 75.0 kDa), which consists of 5.01 mg/mL of l-fucose, 2.02 mg/mL of uronic acids and 1.65 ppm of sulfate, has revealed that at the concentration of 1.0 mg/mL, the G0/G1-phase population in Huh7 hepatocarcinoma cell was increased, accompanied by a decrease in the S phase, highly suggesting that fucoidan may cause the cell cycle arrest at the G0/G1 phase [39]. In a recent study by Zhang et al. [40], it was reported that a high Mw fucoidan HMWF had been extracted from *Cladosiphon navae*. It was then digested with glysidases to acquire LMWF. LMWF was comprised of a digested low-molecular-weight fraction (72%, <500 kDa) and a non-digested fraction that consisted of less than 28% (800 kDa). LMWF consisted mainly of fucose (73%), xylose (12%) and mannose (7%). Their results showed that the LMWF enabled an induction of apoptosis in MDA-MB-231 breast cancer cells, showing a decreased trend in cell viability at the concentrations between 82 and 1640 μg/mL, followed by nuclear shrinkage and fragmentation on further analysis, which was an indication that the cytotoxic effect of LMWF was mediated through apoptotic induction. Kasai et al. [41] performed comparative studies involving apoptosis, where they discovered that type II fucoidan isolated from *F. vesiculosus* (600 kDa), exhibited similar apoptosis-inducing activities through the activation of caspase-8 and -9 in MCF-7 and HeLa cells at concentrations between 10–1000 μg/mL when compared to the low-molecular-weight of a type I fucoidan derivative.

Fucoidan has also been identified as a possible or potential counteracting agent to melanoma. Though therapeutic strategies exist in a form of a single agent or combined therapies, the efficacy depends on a number of factors which include the overall health of the patient, stage of cancer or metastasis and location of melanoma [42]. However, the efficacy of such treatments can somewhat be decreased due to the development of diverse resistance mechanisms. Hence, new therapeutic targets have been urgently needed for melanoma. For instance, *F. vesiculosus* fucoidan (purchased from Sigma) exhibited significant inhibitory effects on the cell proliferation and induction of apoptosis on B16 melanoma cells at 550 μg/mL for 48 h [43]. Such evidence was well executed, which indeed was evidently shown by a strong contention on the side of fucoidan to possess therapeutic potentials.

The efficiency of fucoidan to inhibit cancer cells through activating apoptosis indicates a promising potential as a therapeutic agent. It is also encouraging to note that a couple of clinical studies have been undertaken to develop fucoidan as an anti-cancer therapy by means of combining it with other anti-cancer agents, and the little amount of data accumulated so far seems to be leaning in a positive direction. However, serious considerations in terms of further anti-cancer studies are still required, especially the discrepancy of results between the animal experiments and clinical trials in human. This may be under the influence of how the body absorbs and processes fucoidan, including the way in which fucoidan affects the body. In most cases, such processes are similar across species if not the same, occasionally they can be so different in that a substance may be benign in one species but invalid to the other. Therefore, some sort of a multidisciplinary approach can be considered, with an aim to produce credible results and avoid the discrepancy between animal studies and clinical trials as much as possible. With that being mentioned, a lot of ground still needs to be uncovered, hence it is proper to term fucoidan a ‘potential therapy’ rather than a cure for cancer at this stage until further updates are available.

## 8. Therapeutic Potential against Influenza A Virus

Among viral infections, the flu, with its seasonal outbreaks, has been one of the most problematic viruses worldwide, while medicine and science are in pursuit of amicable solutions. For example, influenza A virus (IAV) has been a formidable pathogen, which has been involved in at least three pandemics since the last century. One of its featured pandemics, which was regarded to be severe, claimed at least 40 million lives worldwide 1918–1919 [44]. In late April of 2009, an influenza A virus i.e., H1N1 [45], was in the limelight causing major awareness and surveillance in countries around the world. Though its prevalence was only for a brief period, its negative impact was rather great [46]. Therefore, scientists have been seeking solutions to eradicate or at least control over IAV. The scientific activities have extended as far as exploring marine sources such as fucoidan.

Recently, a study from Wang et al. [47] was undertaken to inhibit IAV infection by *Kjellmaniella crassifolia* fucoidan (536 kDa, sulfate content 30.1%) targeting the viral neuraminidase and cellular EGFR pathway. The selection of this type of fucoidan was based on one of the other requirements—that the development of anti-IAV drugs must have a high efficacy and minimal or no toxicity [46], hence the study on fucoidan was rather favorable as a consequence that most studies mention that fucoidan has less or no toxicity and is cost-effective compared to possible alternatives. The results revealed that *K. crassifolia* fucoidan blocked IAV infection in vitro with low toxicity, it also exhibited a broad spectrum against IAV and showed a low tendency in the induction of viral resistance, outperforming the regular anti-IAV drug amantadine. *K. crassifolia* fucoidan was able to inactivate virus particles before infection and some stages after adsorption. This was because it could also bind to viral neuraminidase (NA) and inhibit the activity of NA to block the release of IAV. In addition, intranasal administration of *K. crassifolia* fucoidan significantly improved survival and showed a decreased in the viral titers in IAV-infected mice. Sun et al. [48] obtained two *L. japonica* LMWF fractions LF1 and LF2 by degradation, which contained fucose of 42.0% and 30.5%; galactose content of 19.8% and 23.9%; uronic acid content of 5.3% and 3.7%; and sulfate content of 30.7% and 32.5%, respectively. They found a weight-average Mw and number-average Mw to be 7600 and 7300 for LF1 and 3900 and 3700 for LF2, respectively. LF1 and LF2 presented remarkable anti-viral activity in vitro especially in middle and high doses (0.15, 0.3, 0.6, 1.2 and 2.4 mg/mL). In vivo results also indicated that LF1 and LF2 were able to prolong the survival time of virus-infected mice, in addition, it presented an ability to significantly improve the quality of immune organs, immune cell phagocytosis and humoral immunity after intravenous administration of LMWFs (2.5, 5, 10 and 15 mg/kg; a period of 14 days). While such fucoidans present broad-spectrum anti-viral activity, the structure–activity relationship still remains unclear.

Despite the success of the currently available drugs, drug resistance, toxicity and cost still remain unresolved issues in the fight against IAV infection. Hence, the development of novel anti-IAV agents that could be used alone or in conjunction with existing anti-viral drugs is of critical importance. It may be possible that this kind of fucoidan from *K. crassifolia* may be developed further against highly pathogenic strains such as H5N1 or H7N9. In some way, fucoidan also has the potential of being a novel nasal drop or spray for influenza therapy and could serve as prophylaxis in the near future [49].

## 9. The Role of Fucoidan as A Potential Anti-Hepatitis B Virus Treatment

Another virus that presents detrimental effects is the hepatitis B virus (HBV). HBV infects more than 300 million people worldwide and is one of the leading causes of liver disease and liver cancer. The current challenge associated with HBV is the lack of knowledge in terms of predicting the outcome and progression of the HBV infection and an unfulfilled need in understanding the molecular, cellular, immunological and genetic basis of various disease manifestations allied with HBV infection [50]. A number of efforts have been made to improve the immunogenicity of HBV vaccines. There was a study conducted with an aim to investigate on *Fucus evanescens* fucoidan towards HBV vaccination, due to the fact that fucoidan was used as an adjuvant as reported previously. This study found that *Fucus evanescens* fucoidan (130–400 kDa) indeed acted as adjuvants by stimulating the formation of specific antibodies towards the surface of HBV, such as HBs-AG in mice [51]. The mice were immunized with compositions of vaccines contained HBs-AG and fucoidan samples, causing the increase of cytokines (TNF-a, IFN-g and IL-2) in the serum. An increase in the production of such cytokines was detected in the culture of splenocytes stimulated in vitro by fucoidan. A comparison was made that the adjuvant effect of fucoidan and its derivatives was similar to aluminum hydroxide, a traditional licensed adjuvant. Based on a structural analysis, this sample possesses the glycosidic linkage and structural features as follows: 3)-a-l-Fucp (2.4-SO_3_)-(1→4)-a-l-Fucp (2-SO_3_)-(1n. Another investigation showed that *F. vesiculosus* fucoidan was able to inhibit the replication of HBV both in vivo and in vitro [52]. Fucoidan suppressed the HBV replication by the activation of the EKR signal pathway and also enhanced the production of type I interferon via the activation of the host immune system. This newly discovered mechanism suggested another approach, which can be effectively employed to inhibit HBV replication. It was further mentioned that fucoidan alone and/or synergistically can be used to serve as a new therapeutic drug against HBV. The investigation determined that fucoidan significantly inhibited HBV replication in a mouse model in vivo (100 mg of fucoidan at 0, 1, 3, 5 and 7 days post-infection) and in HepG2.2.15 cells in vitro (at the concentration of <200 μg/mL). The results indicated *F. vesiculosus* fucoidan could activate MAPK-ERK1/2 pathway and subsequently promote the expression of IFN-α, causing a decrease in the production of HBV DNA and related proteins. This may suggest the possibility of using fucoidan as an alternative therapeutic strategy against HBV infection. Without a doubt, the consideration of safety and other related mechanisms of fucoidan still require further investigation prior to clinical application.

## 10. Therapeutic Potential as Veterinary Medicine against Canine Distemper Virus (CDV)

Viral infection does not only limit its impact on humans, but also affects wild and domestic animals as well. Though, not a lot of data are available, there are viruses that tend to be lethal to domestic animals e.g., canines [53]. The disadvantage is that therapies or medications suitable for such animals are rather limited and not well documented. This leads to such domestic pets succumbing to certain viruses, especially at their infancy due to a low immune system. For example, canine distemper virus (CDV), a morbillivirus genus member, is a virus that infects quite a range of terrestrial and aquatic carnivores [54]. This infection is characterized by presentations of respiratory and gastrointestinal disorders, followed by immunosuppression and neurological complications in infected hosts [54].

A study was performed to assess the fucoidan’s anti-viral activity against CDV, since this type of virus is quite difficult to treat among canines. However, fucoidan may be part of the solution among the strategies and developments that are currently being undertaken, since the cure is still not available. Trejo-Avila et al. [55] reported that fucoidan extracted from *C. okamuranus* was able to inhibit CDV replication. This extraction contained 90.4% fucoidan and its mean molecular weight was 92.1 kDa, with fucose (38.6%), sulfate (15.9%) and other sugars (23%). It did not only show a reduction in the number of plaques but reduced the size of them as well. This fucoidan enabled an inhibition of CDV replication in Vero cells at an amount of 50% inhibitory concentration (IC50) of 0.1 µg/mL. The selectivity index (SI50) derived was >20,000. This showed that the fucoidan possesses an ability to inhibit the viral infection by interfering in the early steps and also by inhibiting CDV-mediated cell fusion. Therefore, fucoidan may be useful for the development of pharmacological strategies to treat and control CDV infection. Results such as these and many others to follow could be a stepping stone towards inventing the medication or cure against this deadly disease among canines.

## 11. Therapeutic Potential against HIV

A search for a cure towards HIV has been one of the focal points by a number of scientists worldwide. Though, a breakthrough has been noted in terms of the currently available treatment (in the form of anti-retrovirals) to tame the virus. However, a need still exists to eradicate it completely. The challenge with current treatment is related to side effects, especially during the initial introduction. Current treatments can also be cost-prohibitive, though certain countries subsidize affected individuals. This, in turn, places some constraints on governments in terms of exorbitant expenditures in an aim to sustain the lives of the people. Taking this into consideration, research for novel compounds to overcome such limitations is desperately needed.

Their anti-viral activity is dependent on the physical and chemical properties of fucoidan. An investigation found various fucoidans could suppress the infection of Jurkat cells utilizing pseudo-HIV-1 particles which contain envelope proteins of HIV-1 [56]. Therefore, based on the data obtained, the fucoidans (*Saccharina cichorioides* (1.3-α-l-fucan) and *S. japonica* (galactofucan) presented a significant inhibitory effect. This was demonstrated by the efficiency against the lentiviral transduction of fucoidan at rather low concentrations of 0.001–0.05 µg/mL. Another potential anti-HIV agent was *S. swartzii* fucoidan [57]. Bioactive fucoidan fractions (CFF: Crude Fucoidan Fraction; FF1: Fucoidan Fraction 1; FF2: Fucoidan Fraction 2) were isolated from *S. swartzii*. The fucoidan fractions were placed under investigation for anti-HIV-1 properties. Fraction FF2 significantly exhibited anti-HIV-1 activity at concentrations of 1.56 and 6.25 μg/mL which was observed by >50% reduction in HIV-1 p24 antigen levels and reverse transcriptase activity. These fractions were mainly composed of sugars, sulfate and uronic acid, and the total sugar content in the FF1 and FF2 was 61.8% and 65.9%; the sulfate content—19.2% and 24.5%, uronic acid—17.6% and 13.4%, Mw—45 and 30 kDa, respectively. In addition, Thanh et al. [58] concluded that fucoidans derived from the three brown seaweeds, *S. mcclurei* (F_SM_), *S. polycystum* (F_SP_) and *Turbinara ornate* (F_TO_), also displayed similar anti-HIV activities with a mean IC50 ranging from 0.33 to 0.7 g/mL. While the highest sulfate content was found in F_SM_ when compared to the other two fucoidans, and their anti-viral activities were not significantly different, suggesting that sulfate content is not the essential factor for anti-HIV activities of fucoidan. Those fucoidans inhibited HIV-1 infection when they were pre-incubated with the virus but not with the cells, and not after infection, showing that they were able to block the early steps of HIV entry into target cells. Hence, such studies are an indication that fucoidans with a naturally high molecular weight are possibly effective as anti-HIV agents regardless of their backbone. Though, the above results may present a rather positive outlook towards fucoidan as an anti-HIV treatment, more in vitro and in vivo studies are still necessary before proceeding to clinical trials.

## 12. Diabetic and Metabolic Syndrome Control

In recent years, fucoidan has received some intense interest as an agent for treating diabetes and other types of metabolic syndromes (MetS). Fucoidan extracted from *F. vesiculosus* has been known as an α-glucosidase inhibitor that is able to treat diabetes [59]. Among other studies, fucoidan was mentioned to have an ability to attenuate diabetic retinopathy through inhibiting VEGF signaling [60]. Additionally, a report of a low Mw fucoidan was noted to provide protection against diabetic associated symptoms in Goto-Kakizaki rats [61]. Fucoidan also improves glucose tolerance by modulating AMPK signaling and GLUT4 activity [62]. Some studies mention that Fuc-Pg (fucoidan from the sea cucumber *Pearsonothuria graeffei*) with an Mw of 310 kDa can be used as a form of functional food to treat MetS [63]. Fuc-Pg enabled weight reduction in high fat diet-fed mice, it also reduced hyperlipidemia, and protected the liver from steatosis. Concurrently, Fuc-Pg reduced the serum inflammatory cytokines combined with reduced macrophage infiltration into adipose tissue. Furthermore, it was declared that the treatment effect for MetS was primarily related to the 4-*O*-sulfated structure of fucoidan, since it was identified as a tetrasaccharide repeating unit with a backbone of [→3Fuc (2S, 4S) α1→3Fucα1→3Fuc(4S) α1→3Fucα1→]n.

With the rapid development of investigations related to intestinal microbes, in some cases, fucoidan is recognized as a prebiotic to regulate the intestinal ecosystem or microbiome [64]. It promotes the growth of beneficial bacteria which represents a mechanism inhibiting the development of MetS [65]. A report by Parnell et al. [66] showed that prebiotics containing fucoidan can regulate blood glucose and metabolism by providing a beneficial environment for the growth stimulation of probiotics. Cheng et al. [67] also demonstrated that *S. fusiforme* fucoidan (SFF) could modify gut microbiota during the alleviation of streptozotocin-induced hyperglycemia in mice. The yield of SFF was 6.02%., with sulfate content up to 14.55% and the average Mw of 205.8 kDa. This study was done with diabetic mice where after a 6-week administration, SFF impressively decreased the fasting blood glucose, diet and water intake. Additionally, SFF attenuated the pathological changes in the heart and liver tissues, hence, improving liver function. Also, SFF suppressed oxidative stress in STZ-induced diabetic mice which are manifestations associated with MetS. Concurrently, SFF significantly altered the gut microbiota in diabetic mice, what was noted is SFF decreased the relative abundances of the diabetes-related intestinal bacteria, which might be the potential mechanism for relieving the symptoms of diabetes [67].

Though the results indicated in this section seem significant in favor of reversing diabetes and MetS, it should be mentioned that related research is still in progress worldwide. The reason is based on the fact that scientists are still in pursuit of the mechanism which affords fucoidan the ability to reverse diabetes or MetS. Other factors to be considered are short-chain fatty acids (SCFAs), which are known to play a role in providing a conducive environment in the intestine after the oral intake of fucoidan since fucoidan cannot be digested by gastrointestinal enzymes, although their fermentation is regarded to be ideal for gut microbiota to produce SCFAs. Therefore, depending on interest, others would study fucoidan through the disciplines of physiology or pathophysiology in the intestine, some may explore the liver and/or pancreas as they play an integral role in digestion especially in the intestine, some may examine the microbiological context, while also looking into the exact mechanism of fucoidan. The reality is that before fucoidan can be considered as medication or future therapy towards MetS and diabetes, the studies that are still in their early stages would need to be completed. Perhaps a solid future project and/or direction needs to be well established while using a specific type of fucoidan.

## 13. Anti-Coagulant Function

Vascular related disease, such as ischemic heart disease, atherosclerosis and deep vein thrombosis, are still among the leading causes of death worldwide. As reported by the World Health Organization, complications associated with these diseases account for over one-quarter of death throughout the world [68]. In most cases, thrombotic related episodes are usually managed by using anti-coagulant and anti-thrombotic medications, such as heparin, a sulfated glycan belonging to the family of glycosaminoglycans (CAGs) [69]. As it would be anticipated, such therapies tend to present with undesirable and come with severe to moderate side effects that are unavoidable [70]. The side effects linked to heparin include thrombocytopenia [71] and hemorrhagic episodes [72], hence this can limit, defeat or hinder its pharmacological applications.

According to evidence from available studies, it is mentioned that the anti-coagulant activity of fucoidan is dependent on its Mw, sulfate group/total sugar ratio, sulfate position, sulfate degree, and glycoside branching [73]. Chandria et al. [74] discovered that, by preparing *Lessonia vadosa* LMWF using free-radical depolymerization, a better anti-coagulant activity is then exhibited than the naïve fucoidan in a dose-dependent-manner. Jin et al. [75] discovered that fucoidan’s Mw and content of galactose, presented anti-coagulant activity. Documentation based on previous studies indicates that fucoidans with an Mw of 5–100 kDa present as potential anti-coagulants, while fractions greater than 850 kDa are lack of anti-coagulant activity [76]. The fucoidans with an Mw ranging from 10–300 kDa, are regarded as having by far the strongest anti-coagulant activity. In a previous study [77], the authors performed a comparative study of anti-thrombotic and anti-platelet activities of different fucoidans from *L. japonica*, where their results showed that the fucoidan of Mw 27–32 kDa exhibited a much better anti-coagulant and anti-thrombin activity than low molecular weight fucoidans (3.7–7.2 kDa) through intravenous administration. A recent study [78] reported that two dry *Fucus* extracts, DFE-1 and DFE-2, prepared using ultrasound technique, were investigated for their anti-coagulant activity compared to the reference agent heparin. An in vivo experiment on Wistar rats was conducted based on anti-coagulant activities whereby increased blood clotting time was studied—measured by activated partial thromboplastin time (APTT) and prothrombin time (PT). The results indicated that DFE-2 was analogous to the anti-coagulatory effect produced by the reference agent heparin, while DFE-1 showed a weak effect compared to DFE-2 or heparin. The distinct anti-coagulant effect between DFE-1 and DFE-2 might be due to their different physiochemical properties, including fucoidan content, monosaccharide composition and the differences in the contents of polyphenols and sulfate groups. This was indicative that the chemical composition plays an essential role in the anti-coagulant activities of fucoidan.

## 14. Methods

An electronic search was conducted with an aim to identify articles relevant for this literature, from the online database Web of Science from 2000 until 2019. The search included ‘structure of fucoidan’, ‘pharmacokinetics’, ‘fucoidan’, ‘seaweed’, ‘apoptosis’ and ‘anti-viral’. The citation lists were searched manually for other related articles. The strategy used to search is explained in Appendix A.

## 15. Conclusions and Future Perspectives

Fucoidan continues to be promising towards a wide range of potential applications. Since the modern era, reviews and research articles based on the therapeutic applications of fucoidan are on the rise. Therefore, support has been growing in terms of the role that fucoidan could play as a form of therapy. This stems from the fact that brown algae has been used for many years to treat certain ailments in TCM, hence there is a bit of history to be considered. It remains significant or essential that each type of fucoidan necessitates screening and validation for a particular therapeutic activity. A serious consideration on pharmacokinetics, uptake and biodistribution still requires further assessment. Despite the numerous studies done on fucoidan so far, there are still few clinical trials planned and completed. This may be due to a lack of comparative studies, on a specific fucoidan for instance. In most cases, studies are examining different types of fucoidan on different cell lines or animal models. Hence, this makes it difficult to establish a general mechanism that is for a specific type of fucoidan. In addition, there is relatively little information available with regards to the absorption, distribution and excretion of fucoidan. With that being mentioned, the structure of fucoidans still requires attention, since they present a complex structure, even their refined structures are not yet clear or fully understood. The biological activities presented by fucoidan are attractive. However, because most of these studies are carried out by using a relatively crude fucoidan, it is quite difficult to determine the relationships between structure and activity. Future studies based on the clear conformation of fucoidan should assist in establishing a better understanding of their biological properties. This might soon be practically possible, as a consequence of the gradual increase in the availability of detection and measurement techniques. The key element in developing successful therapeutic products is based on the understanding of the chemistry and structural variability of each type of fucoidan. It should be appreciated that some studies indicated in this review are currently undertaking further studies, following up on what they have done previously. Additionally, they also indicate that future studies are currently underway, and will be available soon that is based on the past and present work. This paves the way for what will be known as a ‘tried and tested’ regimen, should it one day be used as therapy.

## Appendix A

### Appendix A.1. Search Strategy

Web of Science:

- TOPIC: (fucoidan)
- TOPIC: (fucoidan) AND TOPIC: (structure)
- TOPIC: (sulfated polysaccharides) AND TOPIC:(structure)
- TOPIC: (sulfated polysaccharides) AND TOPIC:(pharmacokinetics)
- TOPIC: (sulfated polysaccharides) AND TOPIC:(absorption)
- TOPIC: (fucoidan) AND TOPIC: (absorption)
- TOPIC: (fucoidan) AND TOPIC:(pharmacokinetics)
- TOPIC: (fucoidan) AND TOPIC: (preparation)
- TOPIC: (fucoidan) AND TOPIC: (sulfate)
- TOPIC: (fucoidan) AND TOPIC: (species)
- TOPIC: (fucoidan) AND TOPIC: (Fucus vesiculosus)
- TOPIC: (fucoidan) AND TOPIC: (cladosiphon okamuranus)
- TOPIC: (fucoidan) AND TOPIC: (ascophyllum nodosum)
- TOPIC: (fucoidan) AND TOPIC: (fucales)
- TOPIC: (fucoidan) AND TOPIC: (brown algae)
- TOPIC: (fucoidan) AND TOPIC: (cancer)
- TOPIC: (fucoidan) AND TOPIC: (antiviral)
- TOPIC: (fucoidan) AND TOPIC: (synergy)
- TOPIC: (fucoidan) AND TOPIC: (anti-influenza)
- TOPIC: (fucoidan) AND TOPIC: (diabetes)
- TOPIC: (fucoidan) AND TOPIC: (metabolic syndrome)
- TOPIC: (fucoidan) AND TOPIC: (anti-coagulant)
- TOPIC: (fucoidan) AND TOPIC: (anti-canine distemper)
- TOPIC: (Low molecular weight fucoidan)
- TOPIC: (fucoidan) AND TOPIC: (apoptosis)
- TOPIC: (fucoidan) AND TOPIC: (caspase)
- TOPIC: (fucoidan) AND TOPIC: (caspase pathway)
- TOPIC: (fucoidan) AND TOPIC: (intrinsic & extrinsic apoptosis)

### Appendix A.2. Criteria for Considering Studies for This Review

We considered all the subject-related studies for this literature, experimental articles published around 2000–2019 (mainly the summary of the experimental data portrayed, excluding background information covered before year 2000) to have provoked our interest. In addition, relevant articles from the bibliography of articles we found with above-mentioned search terms were selected for this review.
